# Human-Comparable Sensitivity of Large Language Models in Identifying Eligible Studies Through Title and Abstract Screening: 3-Layer Strategy Using GPT-3.5 and GPT-4 for Systematic Reviews

**DOI:** 10.2196/52758

**Published:** 2024-08-16

**Authors:** Kentaro Matsui, Tomohiro Utsumi, Yumi Aoki, Taku Maruki, Masahiro Takeshima, Yoshikazu Takaesu

**Affiliations:** 1 Department of Clinical Laboratory, National Center Hospital National Center of Neurology and Psychiatry Kodaira Japan; 2 Department of Sleep-Wake Disorders, National Institute of Mental Health National Center of Neurology and Psychiatry Kodaira Japan; 3 Department of Psychiatry The Jikei University School of Medicine Tokyo Japan; 4 Graduate School of Nursing Science St. Luke’s International University Tokyo Japan; 5 Department of Neuropsychiatry Kyorin University School of Medicine Tokyo Japan; 6 Department of Neuropsychiatry Akita University Graduate School of Medicine Akita Japan; 7 Department of Neuropsychiatry Graduate School of Medicine University of the Ryukyus Okinawa Japan

**Keywords:** systematic review, screening, GPT-3.5, GPT-4, language model, information science, library science, artificial intelligence, prompt engineering, meta-analysis

## Abstract

**Background:**

The screening process for systematic reviews is resource-intensive. Although previous machine learning solutions have reported reductions in workload, they risked excluding relevant papers.

**Objective:**

We evaluated the performance of a 3-layer screening method using GPT-3.5 and GPT-4 to streamline the title and abstract-screening process for systematic reviews. Our goal is to develop a screening method that maximizes sensitivity for identifying relevant records.

**Methods:**

We conducted screenings on 2 of our previous systematic reviews related to the treatment of bipolar disorder, with 1381 records from the first review and 3146 from the second. Screenings were conducted using GPT-3.5 (gpt-3.5-turbo-0125) and GPT-4 (gpt-4-0125-preview) across three layers: (1) research design, (2) target patients, and (3) interventions and controls. The 3-layer screening was conducted using prompts tailored to each study. During this process, information extraction according to each study’s inclusion criteria and optimization for screening were carried out using a GPT-4–based flow without manual adjustments. Records were evaluated at each layer, and those meeting the inclusion criteria at all layers were subsequently judged as included.

**Results:**

On each layer, both GPT-3.5 and GPT-4 were able to process about 110 records per minute, and the total time required for screening the first and second studies was approximately 1 hour and 2 hours, respectively. In the first study, the sensitivities/specificities of the GPT-3.5 and GPT-4 were 0.900/0.709 and 0.806/0.996, respectively. Both screenings by GPT-3.5 and GPT-4 judged all 6 records used for the meta-analysis as included. In the second study, the sensitivities/specificities of the GPT-3.5 and GPT-4 were 0.958/0.116 and 0.875/0.855, respectively. The sensitivities for the relevant records align with those of human evaluators: 0.867-1.000 for the first study and 0.776-0.979 for the second study. Both screenings by GPT-3.5 and GPT-4 judged all 9 records used for the meta-analysis as included. After accounting for justifiably excluded records by GPT-4, the sensitivities/specificities of the GPT-4 screening were 0.962/0.996 in the first study and 0.943/0.855 in the second study. Further investigation indicated that the cases incorrectly excluded by GPT-3.5 were due to a lack of domain knowledge, while the cases incorrectly excluded by GPT-4 were due to misinterpretations of the inclusion criteria.

**Conclusions:**

Our 3-layer screening method with GPT-4 demonstrated acceptable level of sensitivity and specificity that supports its practical application in systematic review screenings. Future research should aim to generalize this approach and explore its effectiveness in diverse settings, both medical and nonmedical, to fully establish its use and operational feasibility.

## Introduction

Large language models (LLMs) with extensive parameters, honed on substantial textual data, have seen striking advancements recently. Following OpenAI’s third-generation Generative Pre-trained Transformer (GPT-3), LLMs now possess advanced competencies in various natural language processing tasks [1]. Among these, ChatGPT, which is built on GPT-3.5—an iteration that improves upon GPT-3 by integrating both supervised and reinforcement learning techniques—has received particular attention [2,3]. GPT-3.5 has shown exceptional performance in the medical domain, achieving remarkable results on medical licensing examinations across different regions [4]. Furthermore, GPT-4, the successor to GPT-3.5, has exhibited superior performance [5], with its contextual understanding abilities potentially exceeding those of humans [6,7]. Beyond its use for language editing [8,9], both GPT-3.5 and GPT-4 have proven to be effective tools for analyzing and comprehending the abstracts of research papers, offering potential benefits in the screening process for systematic reviews.

Systematic reviews and subsequent meta-analyses bear crucial clinical significance. The screening of titles and abstracts is a crucial step in this process [10-13], often involving more than 1000 papers identified via targeted keyword searches [14]. This screening process can take approximately 1 hour for every 60-120 papers [10], which is a substantial drain on human and time resources. In addition, human error is inevitable in the screening process [15-17], and the number of such errors can increase as the amount of paper to be screened increases possibly due to fatigue and cognitive overload [18,19]. To mitigate this labor-intensive task, attempts have been made to use text mining and machine learning technologies [17,20-29]. Although these methods have successfully reduced the workload, they risk omitting relevant papers, which could result in a high false-negative rate. Specifically, several studies reported the exclusion of records that should have been included in the meta-analysis [20,21,23,29]. Consequently, using machine learning techniques, such as natural language processing, to assist with abstract screening has not yet become widely adopted [14,30]. For systematic reviews, maintaining high sensitivity for studies eligible for full-text assessment, ideally at 100% [10], is crucial if they are to be fully supplanted by an automated process.

With the advanced language-processing capabilities of GPT-3.5 and GPT-4 [2,5], there has been an expectation of achieving higher accuracy in screening processes. Kohandel Gargari et al [31] conducted title and abstract screening using GPT-3.5, but the sensitivity for identifying relevant papers remained at a maximum of 69%, even after attempting various prompt modifications. Khraisha et al [32] explored the use of GPT-4 across different systematic review processes and found that the sensitivity for title and abstract screening ranged between 42% and 50%. Guo et al [33] have also demonstrated the use of GPT-4 in title and abstract screenings; however, the sensitivity for relevant papers was limited to 76%, highlighting the challenge of unintentionally excluding necessary records. Notably, Tran et al [34] used GPT-3.5 for title and abstract screening with rigorous prompt adjustments, achieving a high sensitivity of 97.1% for relevant papers. While this high-sensitivity level might already be suitable for practical use in the systematic review process, its specificity was limited to 37.7% [34].

The aim of this study is to develop a title- and abstract-screening method using GPT-3.5 and GPT-4 that achieves as high a sensitivity as possible. Although the method of using GPT-3.5 by Tran et al [34] achieved high sensitivity for identifying relevant papers, we aim to maintain high sensitivity while also improving specificity through a unique approach that incorporates GPT-4. To achieve this, we subdivided the process of determining inclusion for systematic reviews [11] involving 3 layers of screening. By breaking down the screening process into multiple steps, each addressing a specific aspect, we aimed to optimize the performance of the language models. In this study, we regarded the results of human screening as the gold standard and calculated the sensitivity and specificity of the GPT-3.5 and GPT-4 screening results in comparison with them. Furthermore, we carefully examined the records that were erroneously excluded by GPT-3.5/GPT-4. This examination was conducted to assess the appropriateness of their exclusion.

## Methods

### Language Model Details

GPT-3.5 and GPT-4, LLMs used in this study, are accessible through ChatGPT. However, ChatGPT does not support processing multiple queries against the titles and abstracts of scholarly papers simultaneously. To address this limitation, we leveraged the application programming interfaces (APIs) of GPT-3.5 and GPT-4, known as gpt-3.5-turbo and gpt-4-turbo-preview, respectively [35].

For gpt-3.5-turbo, we used the most current model available, gpt-3.5-turbo-0125. This model could be used at a low cost of US $0.50 per 1M tokens for input and US $1.50 per 1M tokens for output, with approximately 750 tokens corresponding to 1000 words [36]. Similarly, for GPT-4, we used the latest model available, gpt-4-0125-preview, which was available at a cost of US $10.00 per 1M tokens for input and US $30.00 per 1M tokens for output [36].

### Calling the GPT-3.5 and GPT-4 API

In this study, we used Google Spreadsheet and Google Apps Script to interface with the GPT-3.5 and GPT-4 APIs for batch processing. Specifically, we created the “GPT35” function to call the gpt-3.5-turbo-0125 API within Google Spreadsheet. Users can invoke this function by entering “=GPT35([prompt])” into a cell, enabling the intuitive batch processing of multiple titles and abstracts. Similarly, we established the “GPT4” function to access the gpt-4-0125-preview API.

Both the gpt-3.5-turbo-0125 and gpt-4-0125-preview have a parameter called “temperature,” which introduces “variability” in the responses—the higher the temperature, the greater the randomness, with a range between 0 and 2 [37]. As described later in this study, the decision to include or exclude records was delegated to GPT-3.5 and GPT-4. At the preliminary trials, it was observed that setting the temperature above 0 resulted in varying responses from one trial to another. In addition, setting the temperature above 0 can lead to unexpected responses. When instructed to respond with either “E” (for the exclusion) or “I” (for the inclusion), if the temperature is 0, the output will be strictly “E” or “I.” However, if the temperature is above 0, even if it is only 0.1, the response might be, for example, “The answer is ‘E’.” In light of these observations, and primarily to ensure reproducibility, this study fixed the temperature at 0 for all screenings. The Apps Script used in this study is shown in Multimedia Appendix 1.

### Process of Screening and Prompt Engineering

**Figure 1:**
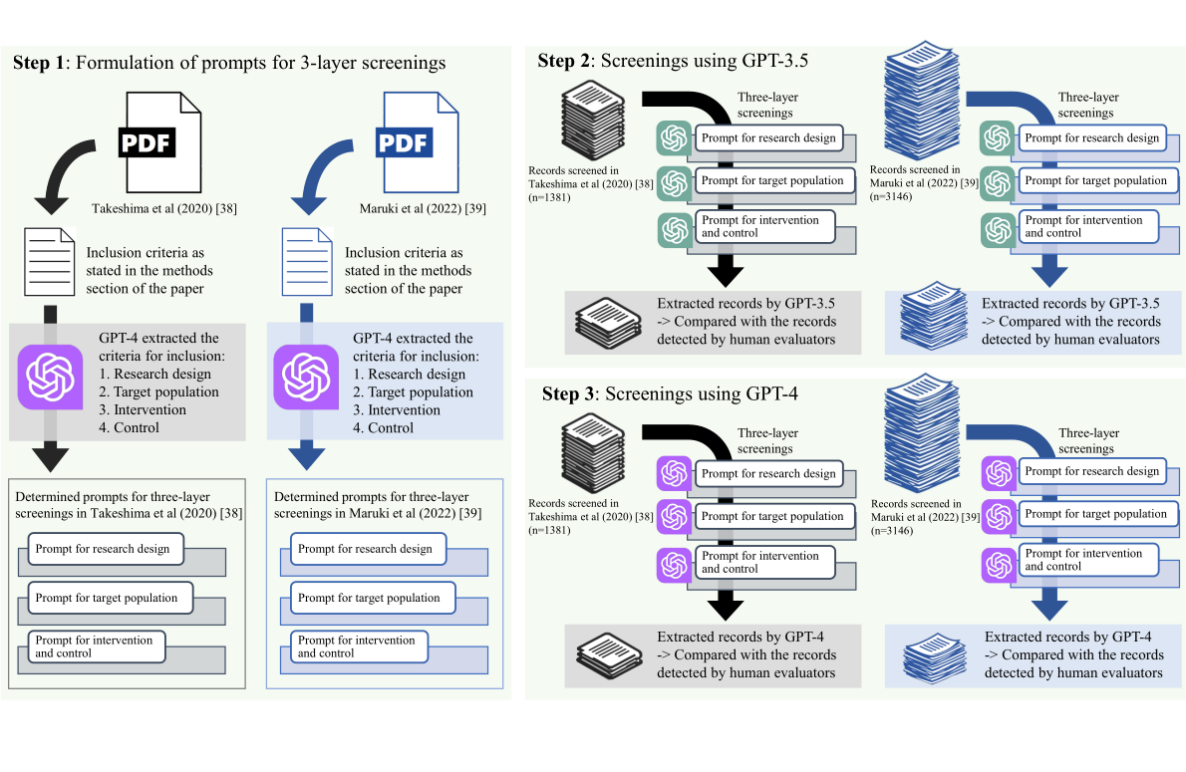
Three-layer screening process using GPT-3.5 and GPT-4 for literature review.

Generally, in a systematic review, a comprehensive examination is conducted on studies that address a relevant clinical question. After a comprehensive literature search is performed to identify all potential studies for review, each record is assessed to determine whether it addresses the clinical question [11]. In this study, we used either GPT-3.5 or GPT-4 to assess the inclusion or exclusion of relevant papers at each of the following three layers: (1) research design, (2) target population, and (3) intervention and control [11]. Records not deemed for exclusion at any of these layers were classified as “included.” We present the workflow of the process we conducted in Figure 1.

The characteristics of the 2 systematic review papers [38,39] used in this study are summarized in Table 1. The first paper by Takeshima et al [38] investigated the efficacy of bright light therapy in patients with bipolar disorder. In this study, the titles and abstracts of a total of 1381 records were initially screened in duplicate, with the task being divided between 2 pairs of independent evaluators. The first pair reviewed the initial 753 records, while the second pair assessed the remaining 628 records. Of these, 30 records were targeted for a full-text assessment, and eventually 6 records (encompassing 6 studies) were selected for meta-analysis. The second paper by Maruki et al [39] verified the difference in therapeutic effects between the usage of 2 types: second-generation antipsychotics (SGAs) and mood stabilizers (MSs), versus the usage of either type alone, targeting patients with bipolar disorder. In this study, the titles and abstracts of a total of 3146 records were initially screened in duplicate, with the screening divided between 2 pairs of evaluators. The first pair reviewed the initial 1694 records, while the second pair evaluated the remaining 1452 records. Of these, 96 records were targeted for a full-text assessment, and eventually 9 records (encompassing 5 studies) were selected for meta-analysis. We used the data on the inclusion or exclusion decisions of each human evaluator made prior to reaching a consensus among evaluators.

**Table 1:** Characteristic of the 2 selected systematic review studies.

	Takeshima et al (2020) [38]	Maruki et al (2022) [39]
Clinical question	Is bright light therapy an effective and safe treatment for managing manic and depressive symptoms in patients with bipolar disorder, and can it also be used as a preventive measure for recurrent mood episodes?	Does the use of second-generation antipsychotics (SGA) or mood stabilizers (MS) as adjunctive therapy improve the efficacy and safety outcomes compared to their use as monotherapy in the treatment of bipolar depression?
Databases	Ovid MEDLINE, Cochrane Central Register of Controlled Trials, Embase, PsycINFO, and ClinicalTrials.gov	PubMed, Cochrane Central Register of Controlled Trials, and Embase
Number of records screened	1381	3146
Number of records for full-text assessment	30	96
Number of records (studies) included in quantitative synthesis	6 (6)	9 (5)

The screening process was divided into three layers: (1) research design, (2) target population, and (3) intervention and control. The prompts for each layer must be specifically tailored to each systematic review. At this point, manual prompt adjustments could lead to issues with reproducibility in future research. Therefore, in this study, we used GPT-4 (gpt-4-0125-preview, temperature=0) to automatically extract the information and generate the content for the prompts related to “research design,” “target population,” “intervention,” and “control.” The prompts used for extraction, along with the content defined for “research design,” “target population,” “intervention,” and “control,” are detailed in Textbox 1. In this study, we extracted information by inserting the text from the “inclusion criteria” paragraph of the Methods section of each paper into the specified location in the prompt (Textbox 1).

The structure of the prompts for each of the 3 layers is shown in Textbox 2. Within these prompts, we specified that if a decision cannot be made, records should be considered potentially eligible for full-text assessment and not excluded. In this study, the information supplied to GPT-3.5 and GPT-4 was limited to the titles and abstracts of the records; details such as authors, their affiliations, or journal names were not included in the prompts.

In the screening process using GPT-3.5 or GPT-4, we initially verified whether the research design of all records satisfied the inclusion criteria. For records not excluded in the first layer, we subsequently confirmed whether the target population aligned with the inclusion criteria. Moreover, for records that were not excluded in the first and second layers, we assessed whether both the intervention and control groups met the inclusion criteria (Figure 1).

**Prompts used for extracting information and the extracted details on research design, target population, intervention, and control from each study.**

- Prompt for extracting information
  #Inclusion criteria
*[The descriptions of the inclusion criteria presented within the Methods section of each paper were inserted here]*
  #Query
  The above is an inclusion criteria of a systematic review paper. Please describe the records included in the meta-analysis in a sentence: 1) research design, 2) target population, 3) intervention, and 4) control. Please list each item without including verbs.
  #Your answer
  1. Research design: [insert your answer here]
  2. Target population: [insert your answer here]
  3. Intervention: [insert your answer here]
  4. Control: [insert your answer here]

- Response to the above prompt
  Inclusion criteria of Takeshima et al (2020) [38], extracted by GPT-4
  1. Research design: Randomized controlled trials (RCTs) at the individual or cluster level, including crossover studies reporting results from the first period.
  2. Target population: Patients with a clinical diagnosis of bipolar disorder (BD), type I or type II.
  3. Intervention: Any kind of light therapy, including 'light therapy,' 'bright light therapy,' 'phototherapy,' or chronotherapy in any intensity and color.
  4. Control: Sham treatment (e.g., low-intensity light, dim red light, or negative ion) or treatment as usual (no light treatment).
  Inclusion criteria of Maruki et al (2022) [39], extracted by GPT-4
  1. Research design: Randomized controlled trials (RCTs) at the individual or cluster level, including crossover studies before crossover
  2. Target population: Participants diagnosed with bipolar I or II depression, including mixed features and/or rapid cycling.
  3. Intervention: Adjunctive therapy with second-generation antipsychotics (SGA) or mood stabilizers (MS) during baseline treatment with SGA or MS.
  4. Control: Adjunctive therapy with a placebo during baseline treatment with second-generation antipsychotics (SGA) or mood stabilizers (MS).

**The structure of the prompts used for each of the 3 layers in the screening process.**

1. Prompt for research design
  #Title and abstract
  Title: [*Title of the record was inserted here*]
  Abstract: [*Abstract of the record was inserted here*]
  #Research design
  [*The ‘research design’ specified in Textbox 1 was inserted here*]
  #Query
  You are a researcher rigorously screening titles and abstracts of scientific papers for inclusion or exclusion in a review paper.
  Does the paper with the above title and abstract meet the specified research design? If yes, highly suspected, or difficult to determine, answer 'I'. If not, answer 'E'.
  #Rules
  You can reply using only 'E' or 'I'.
  #Your answer:
2. Prompt for target population
  #Title and Abstract
  Title: [*Title of the record was inserted here*]
  Abstract: [*Abstract of the record was inserted here*]
  #Target population
  [*The* *‘target population’ specified in Textbox 1 was inserted here*]
  #Query
  You are a researcher rigorously screening titles and abstracts of scientific papers for inclusion or exclusion in a review paper.
  Does the paper with the above title and abstract meet the specified target population? If yes, highly suspected, or difficult to determine, answer ‘I’. If not, answer ‘E’.
  #Rules
  You can reply using only ‘E’ or ‘I’.
  #Your answer:
3. Prompt for intervention and control
  #Title and abstract
  Title: [*Title of the record was inserted here*]
  Abstract: [*Abstract of the record was inserted here*]
  #Intervention
  [*The ‘intervention’ specified in Textbox 1 was inserted here*]
  #Control
  [*The ‘control’ specified in Textbox 1 was inserted here*]
  #Query
  You are a researcher rigorously screening titles and abstracts of scientific papers for inclusion or exclusion in a review paper.
  Does the paper with the above title and abstract meet the specified intervention and control criteria? If yes, highly suspected, or difficult to determine, answer 'I'. If not, answer 'E'.
  #Rules
  You can reply using only 'E' or 'I'.
  #Your answer:

### Data Analysis

In this study, we analyzed the results from human evaluators of systematic review papers, comparing these with the records identified by GPT-3.5 or GPT-4. We considered the records included in the full-text assessment to be correct. We assessed the inclusion or exclusion decisions made by each human evaluator (before consensus was reached) against those determined by GPT-3.5 or GPT-4, focusing on sensitivity and specificity. Sensitivity was defined as the proportion of correctly identified eligible records for full-text assessment by human evaluators, GPT-3.5, or GPT-4. Formally, sensitivity is calculated as follows:

Sensitivity = True positives / (True positives + False negatives)

where:

True positives = Number of records correctly identified as eligible

False negatives = Number of records incorrectly identified as ineligible.

Similarly, specificity was defined as the proportion of correctly identified ineligible records (for full-text assessment) by human evaluators, GPT-3.5, or GPT-4. Formally, specificity is calculated as follows:

Specificity = True negatives / (True negatives + False positives)

where:

True negatives = Number of records correctly identified as ineligible

False Positives = Number of records incorrectly identified as eligible.

For records eligible for full-text assessment but excluded by either GPT-3.5 or GPT-4, we reviewed the title and the abstract to assess whether the exclusion decision was justified. Following this review, we recalculated sensitivity and specificity after adjusting for these justified exclusions. Furthermore, for records that were incorrectly excluded by GPT-3.5 or GPT-4, we conducted a narrative verification of the erroneous judgments by asking each LLM to explain the reasons behind their decisions. We modified the prompt used for screening (Textbox 2) by replacing the “#Rules” statement with “Specify the reason for your answer.” This modification allowed GPT-3.5 or GPT-4 to provide their judgment results along with the underlying reasons.

### Ethical Considerations

This study used only publicly available data from research papers and does not involve human subjects or personal data. Therefore, it does not require a human subject ethics review or exemption.

## Results

### Results on Takeshima et al Paper

**Figure 2:**
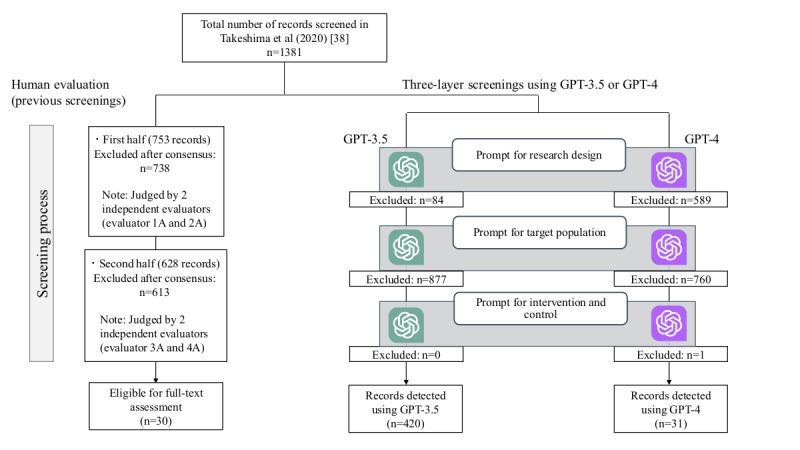
Comparison of 3-layer screening results using GPT-3.5 and GPT-4 with human evaluation for Takeshima et al [38].

Figure 2 [38] shows the number of records excluded by GPT-3.5 and GPT-4 at each layer of research design, target population, and intervention and control, applied to records in the paper by Takeshima et al [38].

GPT-3.5 excluded 84 records at the research design layer, 877 records at the target population layer, and 0 record at the intervention and control layer, ultimately determining 420 out of 1382 records for inclusion. None of the 6 records (including 6 papers) that were included in the meta-analysis were excluded by GPT-3.5. The sensitivity for included records was 0.900 and the specificity was 0.709. Among the eligible records for full-text assessment, GPT-3.5 classified 3 (10.0%) records as excluded. Of these, the exclusion of 2 records by GPT-3.5 was justified, while the remaining 1 (3.3%) record was deemed to require full-text assessment (Table 2). After adjustments for these justified judgments (Multimedia Appendix 2), the sensitivity improved to 0.966 and the specificity remained at 0.710. For the one record that GPT-3.5 determined to be excluded at the target population layer, it was suggested that GPT-3.5 concluded that the record “included both bipolar disorder and unipolar mood disorder, which did not match the selection criteria.”

**Table 2:** Records for full-text assessment in the study by Takeshima et al [38] paper but were excluded by GPT-3.5 and GPT-4.

		Number of excluded records on each layer (number of those not justified)		
		Research design	Target population	Intervention and control
**Number of records eligible for full-text assessment (n=30)**				
	Excluded by GPT-3.5	0	3 (1)^a^	0
	Excluded by GPT-4	4 (1)^a^	2 (0)^a^	0

^a^Number of records for which exclusion was not justified.

GPT-4 excluded 589 records at the research design layer, 760 records at the target population layer, and 1 record at the intervention and control layer, ultimately determining 31 out of 1381 records for inclusion. None of the 6 records (including 6 papers) that were included in the meta-analysis were excluded by GPT-4. The sensitivity for included records was 0.806 and the specificity was 0.996. Among the eligible records for full-text assessment, GPT-4 classified 6 (20.0%) records as excluded. Of these, the exclusion of 5 records by GPT-4 was justified, while the remaining 1 (3.3%) record was considered to require full-text assessment (Table 2). After adjustments for these justified judgments (Multimedia Appendix 2), the sensitivity improved to 0.962 and the specificity remained at 0.996. GPT-4 included all 6 records (including 6 papers) that were included in the meta-analysis. For the one record that GPT-4 judged to be excluded at the research design layer, it was revealed that GPT-4 deduced that “although this study mentioned registration in an RCT, it investigated the associations between sleep, physical activity, and circadian rhythm indicators” (from the perspective of whether to include the study in the meta-analysis, GPT-4’s judgment is likely to be correct; however, considering the purpose of the initial screening, we determined that it would be appropriate to include the study).

### Results of the Paper by Maruki et al

**Figure 3:**
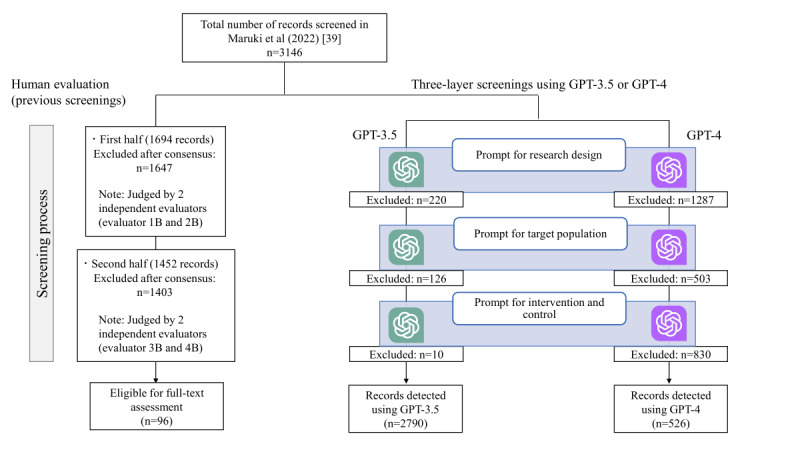
Comparison of 3-layer screening results using GPT-3.5 and GPT-4 with human evaluation for Maruki et al [39].

Figure 3 [39] shows the number of records excluded by GPT-3.5 and GPT-4 at each layer of research design, target population, and intervention and control, applied to records in the Maruki et al [39] paper.

GPT-3.5 excluded 220 records at the research design layer, 126 records at the target population layer, and 10 records at the intervention and control layer, ultimately determining 2790 out of 3146 records for inclusion. None of the 9 records (including 9 papers) that were included in the meta-analysis were excluded by GPT-3.5. The sensitivity for included records was 0.958 and the specificity was 0.116. Among the eligible records for full-text assessment, GPT-3.5 classified 4 (4.2%) records as excluded. None of these records’ exclusion by GPT-3.5 was justified, and all were considered to require full-text assessment (Table 3 and Multimedia Appendix 2). For the 2 records that GPT-3.5 inferred to be excluded at the research design layer, it was revealed that GPT-3.5 determined that “although they were RCTs, either the individual or cluster level was not specified” for both records. For the 2 records that GPT-3.5 deemed to be excluded at the target population layer, it was suggested that GPT-3.5 surmised that “although the records involved bipolar disorder, they did not match the selection criteria due to the presence of comorbidities (one record had generalized anxiety disorder, and the other had alcohol dependence).”

**Table 3:** Records for full-text assessment in the paper by Maruki et al [39] but were excluded by GPT-3.5 and GPT-4.

		Number of excluded records on each layer (number of those not justified)		
		Research design	Target population	Intervention and control
			
**Number of records eligible for full-text assessment (n=96)**				
	Excluded by GPT-3.5	2 (2)^a^	2 (2)^a^	0
	Excluded by GPT-4	5 (0)^a^	2 (1)^a^	5 (3)^a^

^a^Number of records for which exclusion was not justified.

GPT-4 excluded 1287 records at the research design layer, 503 records at the target population layer, and 830 records at the intervention and control layer, ultimately determining 526 out of 3146 records for inclusion. None of the 9 records (including 9 papers) that were included in the meta-analysis were excluded by GPT-4. The sensitivity for included records was 0.875 and the specificity was 0.855. Among the eligible records for full-text assessment, GPT-4 classified 12 (12.5%) records as excluded. Of these, the exclusion of 8 records by GPT-4 was justified, while the remaining 4 (4.2%) records were considered to require full-text assessment (Table 3). After adjustments for these justified judgments (Multimedia Appendix 2), the sensitivity improved to 0.943 and the specificity remained at 0.855. “For the one record that GPT-4 determined to be excluded at the target population layer, it was suggested that GPT-4 inferred that ‘although the record involved bipolar disorder, it did not match the selection criteria due to the presence of a comorbidity (alcohol dependence).’ For the three records that GPT-4 judged to be excluded at the Intervention and control layer, in each case, GPT-4 cited the reason for exclusion as ‘the intervention criteria are the addition of either SGA or MS to SGA or MS, but this study does not mention the use of SGA.’”

In the list used in the paper by Maruki et al [39], there were a total of 355 records where part of the title and abstract were corrupted into irrelevant Chinese characters (eg, “This was an eight窶陣eek, open窶人abel, prospective study”). Despite these errors, all cases could be appropriately discerned, likely due to the context-sensitive judgment capability of GPT-3.5 and GPT-4.

### Comparison of GPT-3.5, GPT-4, and Human Evaluators

Both the study by Takeshima et al [38] and the study by Maruki et al [39] involved 2 individuals conducting screening for the initial segment, while a different set of 2 individuals was responsible for the screening of the latter segment. The sensitivity and specificity of human evaluators and GPT-3.5 and GPT-4 for each segment are shown in Table 4. The adjusted results, in cases where the exclusion of GPT-3.5 or GPT-4 was justified, are shown in the numbers within parentheses (Table 4).

**Table 4:** Comparison of evaluation metrics: GPT-3.5, GPT-4, and human evaluators.

Screenings on Takeshima et al (2020) [38]		Human evaluators					LLMs^a^	
		1A	2A	3A	4A	GPT-3.5		GPT-4
**Initial segment (n=753)**								
	Sensitivity	1.000	0.867	—^b^	—	0.800 (0.929)^c^		0.688 (1.000)^c^
	Specificity	0.995	0.996	—	—	0.702 (0.704)^c^		0.997 (0.997)^c^
**Latter segment (n=628)**								
	Sensitivity	—	—	1.000	1.000	1.000 (1.000)^c^		0.933 (0.933)^c^
	Specificity	—	—	1.000	0.997	0.718 (0.718)^c^		0.993 (0.993)^c^
Screenings on Maruki et al (2022) [39]		Human evaluators	Human evaluators	Human evaluators	Human evaluators	LLMs		LLMs
Screenings on Maruki et al (2022) [39]		1B	2B	3B	4B	GPT-3.5		GPT-4
**Initial segment (n = 1694)**								
	Sensitivity	0.766	0.979	—	—	0.936		0.872 (0.952)^c^
	Specificity	0.998	0.998	—	—	0.129		0.886 (0.886)^c^
**Latter segment (n=1452)**								
	Sensitivity	—	—	0.776	0.939	0.980		0.878 (0.935)^c^
	Specificity	—	—	0.999	0.999	0.100		0.818 (0.819)^c^

^a^LLMs: large language models.

^b^Not applicable.

^c^Values after adjusting for cases where exclusion was justified.

### Time and Cost Required for Screenings

In our Google Spreadsheet setup, both GPT-3.5 and GPT-4 managed to process approximately 110 records per minute across each of the 3 layers. Consequently, the estimated ideal completion time was between 20 and 30 minutes for the study by Takeshima et al [38], and between 60 and 80 minutes for the study by Maruki et al [39]. However, in practice, due to errors with the Google Spreadsheet and API, the screening process took about 1 hour for the study by Takeshima et al [38] and about 2 hours in total for the study by Maruki et al [39]. Furthermore, due to daily API call limits, the work had to be spread out over 3 days. The screening for these 2 studies incurred a total cost of US $59, with US $4 for calls to GPT-3.5 and US $55 for calls to GPT-4.

## Discussion

### Principal Findings

This study demonstrates the use of a 3-layer screening method using GPT-3.5 and GPT-4 for title and abstract screenings in systematic reviews, highlighting its remarkable speed and sensitivity comparable with that of human evaluators. However, GPT-3.5 demonstrated low specificity for relevant records, rendering it less practical. In contrast, the use of GPT-4 showed both high sensitivity and specificity, particularly where adjustments for justified exclusions led to an improvement in sensitivity. Although achieving 100% sensitivity remained unattainable, a 3-layer screening method with GPT-4 may potentially be practical for use in the systematic review process and can reduce human labor.

Previous research demonstrating the effectiveness of automated screening using text mining has encountered sensitivity issues [20-29]. Specifically, the exclusion of important studies that should have been included in their meta-analysis [20,21,23,29], a limitation not observed in our approach, hampered their application to clinical practice. False negatives in machine learning–based screening can arise from several factors: complexity in research design, characteristics of the target demographic, types of interventions, complexity in selection criteria, a significant scarcity of relevant records within the data set (leading to data imbalance), and inconsistency in the terminology used for judgment [21,23,29]. Our method using GPT-3.5 or GPT-4 was able to address issues related to data set imbalance and terminology inconsistency, as we used the same prompt across records, and assess the inclusion or exclusion one by one. In addition, previous text mining screenings may not have effectively addressed garbled text, such as “open-label” mistakenly appearing as “open窶人abel” [40], an issue that LLMs can potentially mitigate through their attention mechanisms [41]. Moreover, the outstanding knowledge base of GPT-4 [6,7] likely helped address the complexity in research design, target demographics, and intervention, as well as selection criteria—areas where GPT-3.5 might have fallen short. These distinctions possibly account for the notable differences in specificity observed between GPT-3.5 and GPT-4. Recently, Guo et al [33] conducted title and abstract screening using GPT-4. Their approach diverges from our 3-layer method; it integrated inclusion and exclusion criteria within the context, generating decisions and reasoning through a single prompt. While we believe that our 3-layer method could potentially offer greater sensitivity than theirs, it remains difficult to definitively assert a significant improvement in sensitivity over the method by Guo et al [33], given the limited sample size and the differences in data sets. Tran and colleagues’ approach [34], despite using GPT-3.5, demonstrated remarkable sensitivity. It is important to note, however, that the manual creation of their highly effective prompt raises questions regarding its replicability and broader applicability.

Both human-conducted and LLM-conducted systematic reviews have their inherent pitfalls. Errors made by humans are inevitable, with their accuracy estimated to be around 10% [15], and slightly higher for false exclusions, at approximately 13%-14% [16,17]. These values represent the performance of experts in the relevant field, and the accuracy may be lower for individuals with less expertise or shallow screening experience; therefore, guidelines have recommended piloting and training the abstract screening team [12]. In this study, we observed that human evaluation in the paper by Takeshima et al [38] exhibited slightly more false negatives than that in the paper by Maruki et al [39]. Although the reasons for the judgment discrepancies were not investigated in this study’s data set, they may be attributed to the larger volume of records screened [14] and the potentially more complex and challenging research question in the paper by Maruki et al [39]. Using 2 reviewers to screen records can significantly lower the likelihood of false negatives [16] and has been recommended [11,13]. Yet, simultaneously, there has been a case that the systematic review screenings, albeit rare, are conducted by a single reviewer, because of time constraints [13,42]. Hence, the unavoidable errors and substantial time and effort required for screening represent significant drawbacks of human screening in systematic reviews [10,13].

Conversely, methods using LLMs also present several drawbacks. One primary concern is their susceptibility to misinformation and quality issues inherent in their training data [43]. Notably, in this study, the specificity of the GPT-3.5 screenings in Maruki et al [39] paper was markedly low. While the causes are not definitive, this may be attributed to an insufficient understanding of bipolar disorder, MSs, and second-generation antipsychotics. Tran and colleagues [34] incorporated relevant knowledge into their manually created prompts; it might have enhanced sensitivity but not specificity; and this could also be due to GPT-3.5’s knowledge limitations. Furthermore, the decision-making processes of LLMs lack transparency, making them difficult to interpret [43]. This lack of interpretability is compounded by the “grounding problem,” where LLMs struggle to grasp concrete facts and real-world scenarios due to their lack of real-world experiences and sensory input [1,44]. We attempted to verify incorrectly excluded records by querying GPT-3.5 and GPT-4 with the original screening prompts, their responses, and justifications. Our findings revealed that GPT-3.5’s lower accuracy was primarily due to a lack of knowledge about the target domain, while GPT-4’s incorrect exclusions were mainly due to misinterpretations of the inclusion criteria. These findings highlight the ongoing challenges in understanding and interpreting the decision-making processes of LLMs. Although GPT-4 demonstrates advancements in comprehension, factuality, specificity, and inference, it is still more susceptible to factual errors [45]. In addition, it has been suggested that LLMs’ accuracy diminishes with longer prompts [46]; lengthy abstracts might have contributed to decreased accuracy in decision-making. A potential future risk is that the normalization of AI-based judgments could result in the oversight of human expert verification, potentially diminishing the quality of systematic reviews.

On the positive side, compared with the human screening time reported in previous studies [10], our method enabled remarkably faster screening. Although our approach uses a 3-layer structure, which might seem time-consuming at first glance, by limiting GPT-3.5/GPT-4 responses to “E” (Exclude) or “I” (Include), we efficiently screened a large volume of records in batch. Unlike humans, LLMs do not experience fatigue and subsequent decline in performance; moreover, they are presumed to have better reproducibility in their judgments. While using GPT-4’s API comes with associated costs [36], the increased efficiency compared with human effort more than compensates for these expenses. Using LLMs for title and abstract screening could also enable screening a much larger number of records, previously deemed impractical due to time limitations. Our 3-layer method using GPT-4 exhibits high sensitivity and a useful level of specificity and yet opportunities for further refinement exist. Future studies could enhance accuracy through methods such as optimizing prompts [47] and integrating multiple LLMs for decision assessment [48], which may contribute to higher precision. In the meantime, swift advancements in LLM technology are set to continuously evolve; future breakthroughs in LLMs may readily overcome our current challenges—possibly, only by a simple prompt.

### Limitations

This study has some limitations. First, the 2 systematic reviews used in this investigation [38,39] were confined to clinical studies within psychiatry, limiting the generalizability of our findings. In addition, the sample size was small, and the investigation remained exploratory, with the results lacking statistical substantiation. Future studies should aim to replicate these findings across a broader range of medical fields and specialized domains to enhance their applicability and reliability. Second, the artificial intelligence industry is progressing rapidly, with information becoming obsolete within a matter of months or even weeks. The models we used in this study, gpt-3.5-turbo-0125 and gpt-4-0125-preview, are currently the most up-to-date. However, updates to these models might alter screening outcomes. Third, to ensure consistency in our findings, we set the temperature parameter to 0. However, a temperature of 0 does not always guarantee absolute uniformity in output sentences [35]. However, our observations indicate no variation in results across multiple tests with the same model in this study. Fourth, this study did not investigate the discrepancies in screening results between GPT-3.5 and GPT-4, nor did it examine the impact of prompt variations on performance. In addition, this research did not directly compare the performance of the proposed approach with existing systematic literature review strategies. Furthermore, this study was not designed to explore the risks associated with using LLMs for screening purposes. Finally, gpt-3.5-turbo-0125’s training data include information up to September 2021, whereas gpt-4-0125-preview’s training data extend to December 2023 [35]. Consequently, the systematic review paper by Takeshima et al [38] might have been incorporated into GPT-3.5’s training data set, with both systematic review papers possibly included in GPT-4’s data set. Nevertheless, as the study’s prompts did not explicitly reference these reviews, we consider that their impact is minimal.

### Conclusions

We developed a practical screening method using GPT-3.5 and GPT-4 in the title- and abstract-screening process of systematic reviews. Our 3-layer method not only achieved better sensitivity for relevant records than previous machine learning–based screening methods [20,21,23,29] but also demonstrated a remarkable potential to reduce human reviewers’ workload significantly. Although GPT-3.5 showed lower specificity, which may limit its applicability, the use of GPT-4 within our method yielded sensitivity comparable with human evaluators, making it suitable for use in systematic review screenings. Despite the focus on psychiatric fields and the small sample size of our study, our findings highlight the potential for broader application. We emphasize the importance of further validation across multiple domains to establish a universal screening methodology. Concurrently, developing more effective approaches in response to the advancing capabilities of LLMs is warranted in future research.

## Appendix

Multimedia Appendix 1 Script for the Google Spreadsheet.

Multimedia Appendix 2 Records eligible for full paper screening but excluded by GPT-3.5 or GPT-4.

## Abbreviations

API: application programming interface
GPT: Generative Pre-trained Transformer
LLM: large language model
MS: mood stabilizers
SGA: second-generation antipsychotics

## References

[1] Brown TB, Mann B, Ryder N, Subbiah M, Kaplan JD, Dhariwal P, Neelakantan A, Shyam P, Sastry G, Askell A, Agarwal S, Herbert-Voss A, Krueger G, Henighan T, Child R, Ramesh A, Ziegler DM, Wu J, Winter C, Hesse C, Chen M, Sigler E, Litwin M, Gray S, Chess B, Clark J, Berner C, McCandlish S, Radford A, Sutskever I, Amodei D (2020). Language models are few-shot learners. Adv Neural Inf Process Syst.

[2] Ouyang L, Wu J, Jiang X, Almeida D, Wainwright CL, Mishkin P, Zhang C, Agarwal S, Slama K, Ray A, Schulman J, Hilton J, Kelton F, Miller L, Simens M, Askell A, Welinder P, Christiano P, Leike J, Lowe R (2022). Training language models to follow instructions with human feedback. Adv Neural Inf Process Syst.

[3] Introducing ChatGPT.

[4] Levin G, Horesh N, Brezinov Y, Meyer R (2024). Performance of ChatGPT in medical examinations: a systematic review and a meta-analysis. BJOG.

[5] GPT-4.

[6] Bojic L, Kovacevic P, Cabarkapa M (2023). GPT-4 surpassing human performance in linguistic pragmatics. arXiv. Preprint posted online.

[7] Eriksen AV, Möller S, Ryg J (2023). Use of GPT-4 to diagnose complex clinical cases. NEJM AI.

[8] Kim SG (2023). Using ChatGPT for language editing in scientific articles. Maxillofac Plast Reconstr Surg.

[9] Matsui K, Koda M, Yoshida K (2023). Implications of nonhuman "Authors". JAMA.

[10] Lefebvre C, Glanville J, Briscoe S, Littlewood A, Marshall C, Metzendorf MI, Noel-Storr A, Rader T, Shokraneh F, Thomas J, Wieland LS (2019). Searching for and selecting studies. Cochrane Handbook for Systematic Reviews of Interventions.

[11] Higgins JPT, Thomas J, Chandler J, Cumpston M, Li T, Page MJ (2019). Cochrane Handbook for Systematic Reviews of Interventions.

[12] Polanin JR, Pigott TD, Espelage DL, Grotpeter JK (2019). Best practice guidelines for abstract screening large‐evidence systematic reviews and meta‐analyses. Res Synth Methods.

[13] Page MJ, Moher D, Bossuyt PM, Boutron I, Hoffmann TC, Mulrow CD, Shamseer L, Tetzlaff JM, Akl EA, Brennan SE, Chou R, Glanville J, Grimshaw JM, Hróbjartsson A, Lalu MM, Li T, Loder EW, Mayo-Wilson E, McDonald S, McGuinness LA, Stewart LA, Thomas J, Tricco AC, Welch VA, Whiting P, McKenzie JE (2021). PRISMA 2020 explanation and elaboration: updated guidance and exemplars for reporting systematic reviews. BMJ.

[14] O'Hearn K, MacDonald C, Tsampalieros A, Kadota L, Sandarage R, Jayawarden SK, Datko M, Reynolds JM, Bui T, Sultan S, Sampson M, Pratt M, Barrowman N, Nama N, Page M, McNally JD (2021). Evaluating the relationship between citation set size, team size and screening methods used in systematic reviews: a cross-sectional study. BMC Med Res Methodol.

[15] Wang Z, Nayfeh T, Tetzlaff J, O'Blenis P, Murad MH (2020). Error rates of human reviewers during abstract screening in systematic reviews. PLoS One.

[16] Gartlehner G, Affengruber L, Titscher V, Noel-Storr A, Dooley G, Ballarini N, König F (2020). Single-reviewer abstract screening missed 13 percent of relevant studies: a crowd-based, randomized controlled trial. J Clin Epidemiol.

[17] Wilson E, Cruz F, Maclean D, Ghanawi J, McCann SK, Brennan PM, Liao J, Sena ES, Macleod M (2023). Screening for in vitro systematic reviews: a comparison of screening methods and training of a machine learning classifier. Clin Sci (Lond).

[18] Bannach-Brown A, Przybyła P, Thomas J, Rice ASC, Ananiadou S, Liao J, Macleod MR (2019). Machine learning algorithms for systematic review: reducing workload in a preclinical review of animal studies and reducing human screening error. Syst Rev.

[19] Cierco Jimenez R, Lee T, Rosillo N, Cordova R, Cree IA, Gonzalez A, Indave Ruiz BI (2022). Machine learning computational tools to assist the performance of systematic reviews: a mapping review. BMC Med Res Methodol.

[20] Shemilt I, Simon A, Hollands GJ, Marteau TM, Ogilvie D, O'Mara-Eves A, Kelly MP, Thomas J (2014). Pinpointing needles in giant haystacks: use of text mining to reduce impractical screening workload in extremely large scoping reviews. Res Synth Methods.

[21] Rathbone J, Hoffmann T, Glasziou P (2015). Faster title and abstract screening? Evaluating Abstrackr, a semi-automated online screening program for systematic reviewers. Syst Rev.

[22] Olofsson H, Brolund A, Hellberg C, Silverstein R, Stenström K, Österberg M, Dagerhamn J (2017). Can abstract screening workload be reduced using text mining? User experiences of the tool Rayyan. Res Synth Methods.

[23] Gates A, Johnson C, Hartling L (2018). Technology-assisted title and abstract screening for systematic reviews: a retrospective evaluation of the Abstrackr machine learning tool. Syst Rev.

[24] Gartlehner G, Wagner G, Lux L, Affengruber L, Dobrescu A, Kaminski-Hartenthaler A, Viswanathan M (2019). Assessing the accuracy of machine-assisted abstract screening with DistillerAI: a user study. Syst Rev.

[25] Gates A, Gates M, Sebastianski M, Guitard S, Elliott SA, Hartling L (2020). The semi-automation of title and abstract screening: a retrospective exploration of ways to leverage abstrackr's relevance predictions in systematic and rapid reviews. BMC Med Res Methodol.

[26] Hamel C, Kelly SE, Thavorn K, Rice DB, Wells GA, Hutton B (2020). An evaluation of DistillerSR's machine learning-based prioritization tool for title/abstract screening—impact on reviewer-relevant outcomes. BMC Med Res Methodol.

[27] Reddy SM, Patel S, Weyrich M, Fenton J, Viswanathan M (2020). Comparison of a traditional systematic review approach with review-of-reviews and semi-automation as strategies to update the evidence. Syst Rev.

[28] Pham B, Jovanovic J, Bagheri E, Antony J, Ashoor H, Nguyen TT, Rios P, Robson R, Thomas SM, Watt J, Straus SE, Tricco AC (2021). Text mining to support abstract screening for knowledge syntheses: a semi-automated workflow. Syst Rev.

[29] Valizadeh A, Moassefi M, Nakhostin-Ansari A, Hosseini Asl SH, Saghab Torbati M, Aghajani R, Maleki Ghorbani Z, Faghani S (2022). Abstract screening using the automated tool rayyan: results of effectiveness in three diagnostic test accuracy systematic reviews. BMC Med Res Methodol.

[30] O'Connor AM, Tsafnat G, Thomas J, Glasziou P, Gilbert SB, Hutton B (2019). A question of trust: can we build an evidence base to gain trust in systematic review automation technologies?. Syst Rev.

[31] Kohandel Gargari O, Mahmoudi MH, Hajisafarali M, Samiee R (2024). Enhancing title and abstract screening for systematic reviews with GPT-3.5 turbo. BMJ Evid Based Med.

[32] Khraisha Q, Put S, Kappenberg J, Warraitch A, Hadfield K (2024). Can large language models replace humans in systematic reviews? Evaluating GPT-4's efficacy in screening and extracting data from peer-reviewed and grey literature in multiple languages. Res Synth Methods.

[33] Guo E, Gupta M, Deng J, Park YJ, Paget M, Naugler C (2024). Automated paper screening for clinical reviews using large language models: data analysis study. J Med Internet Res.

[34] Tran VT, Gartlehner G, Yaacoub S, Boutron I, Schwingshackl L, Stadelmaier J, Sommer I, Alebouyeh F, Afach S, Meerpohl J, Ravaud P (2024). Sensitivity and specificity of using GPT-3.5 turbo models for title and abstract screening in systematic reviews and meta-analyses. Ann Intern Med.

[35] Models.

[36] Pricing.

[37] API Reference.

[38] Takeshima M, Utsumi T, Aoki Y, Wang Z, Suzuki M, Okajima I, Watanabe N, Watanabe K, Takaesu Y (2020). Efficacy and safety of bright light therapy for manic and depressive symptoms in patients with bipolar disorder: a systematic review and meta-analysis. Psychiatry Clin Neurosci.

[39] Maruki T, Utsumi T, Takeshima M, Fujiwara Y, Matsui M, Aoki Y, Toda H, Watanabe N, Watanabe K, Takaesu Y (2022). Efficacy and safety of adjunctive therapy to lamotrigine, lithium, or valproate monotherapy in bipolar depression: a systematic review and meta-analysis of randomized controlled trials. Int J Bipolar Disord.

[40] Benchimol J, Kazinnik S, Saadon Y (2022). Text mining methodologies with R: An application to central bank texts. Machine Learn with Appl.

[41] Vaswani A, Shazeer N, Parmar N, Uszkoreit J, Jones L, Gomez AN, Kaiser L, Polosukhin I (2017). Attention is all you need. Adv Neural Inf Process syst.

[42] Nussbaumer-Streit B, Mayr V, Dobrescu AI, Chapman A, Persad E, Klerings I, Wagner G, Siebert U, Christof C, Zachariah C, Gartlehner G (2020). Quarantine alone or in combination with other public health measures to control COVID-19: a rapid review. Cochrane Database Syst Rev.

[43] Wang B, Xie Q, Pei J, Chen Z, Tiwari P, Li Z, Fu J (2023). Pre-trained language models in biomedical domain: a systematic survey. ACM Comput Surv.

[44] Mollo DC, Millière R (2023). The vector grounding problem. arXiv. Preprint posted online.

[45] Zheng S, Huang J (2023). Chang KC-C. why does chatgpt fall short in providing truthful answers. arXiv. Preprint posted online.

[46] Levy M, Jacoby A, Goldberg Y (2024). Same task, more tokens: the impact of input length on the reasoning performance of large language models. arXiv. Preprint posted online.

[47] Giray L (2023). Prompt engineering with ChatGPT: a guide for academic writers. Ann Biomed Eng.

[48] Li J, Zhang Q, Yu Y, Fu Q, Ye D (2024). More agents is all you need. arXiv. Preprint posted online.

