# Supplementary material for: Human-Comparable Sensitivity of Large Language Models in Identifying Eligible Studies Through Title and Abstract Screening: 3-Layer Strategy Using GPT-3.5 and GPT-4 for Systematic Reviews
Source: J Med Internet Res. 2024 Aug 16;26:e52758. doi: 10.2196/52758 (PMC11364944; doi:10.2196/52758)
Supplement: Multimedia Appendix 1 [file jmir_v26i1e52758_app1.docx]

Multimedia Appendix 1. Script for the Google Spreadsheet

var apiKey = "*******************************************"; // API Key

// Configuration for gpt-3.5-turbo-0125

var apiUrl = "https://api.openai.com/v1/chat/completions";

var model = "gpt-3.5-turbo-0125";

// Function for gpt-3.5-turbo-0125

function GPT35(prompt) {

    return callGptApi(prompt, model);

}

// Configuration for gpt-4-0125-preview

var modelGPT4 = "gpt-4-0125-preview";

// Function for gpt-4-0125-preview

function GPT4(prompt) {

    return callGptApi(prompt, modelGPT4);

}

function callGptApi(prompt, model) {

    // Setting up the messages

    let messages = [{role: "user", content: prompt}];

    temperature = 0; // Defines randomness in the AI's responses

    maxTokens = 2000; // The maximum length of the generated text

    // Configuration for the API request

    const payload = {

        model: model,

        messages: messages,

        temperature: temperature,

        max_tokens: maxTokens,

    };

    const options = {

        method: "POST", // Method of the request

        headers: {

            "Authorization": "Bearer "+apiKey, // Inserting the API key in the request header

            "Content-Type": "application/json" // Type of content

        },

        payload: JSON.stringify(payload), // Turning the payload object into a string

    }

    // Sending the request and receiving the response

    const response = UrlFetchApp.fetch(apiUrl, options);

    var responseText = response.getContentText(); // Getting the text content from the response

    var json = JSON.parse(responseText); // Parsing the text into a JSON object

    return json.choices[0].message.content.trim(); // Returning the first generated message content

}
